# Cooperation between AlphavBeta3 integrin and the fibroblast growth factor receptor enhances proliferation of Hox-overexpressing acute myeloid leukemia cells

**DOI:** 10.18632/oncotarget.10189

**Published:** 2016-06-20

**Authors:** Chirag A. Shah, Ling Bei, Hao Wang, Jessica K. Altman, Leonidas C. Platanias, Elizabeth A. Eklund

**Affiliations:** ^1^ The Feinberg School of Medicine and Robert H. Lurie Comprehensive Cancer Center of Northwestern University, Chicago, IL, USA; ^2^ Jesse Brown Veteran's Administration Medical Center, Chicago, IL, USA

**Keywords:** transcription factor, integrin, leukemia, myeloid cell, homeobox, fibroblast growth factor

## Abstract

A poor prognosis subtype of acute myeloid leukemia (AML) is characterized by increased expression of a set of homeodomain (HD) transcription factors, including HoxA9, HoxA10 and Cdx4. This encompasses AML with *MLL1* gene translocations, because Mll1-fusion proteins aberrantly activate *HOX* transcription. We previously identified *FGF2* (Fibroblast Growth Factor 2) as a target gene for HoxA9 and HoxA10 that was indirectly activated by Mll-Ell (an Mll1-fusion protein). Autocrine stimulation of Mll-Ell^+^ myeloid progenitor cells by Fgf2 stabilized βcatenin and increased expression of βcatenin target genes, including *CDX4*. Since *HOXA9* and *HOXA10* are Cdx4 target genes, Fgf2 indirectly augmented direct effects of Mll-Ell on these genes. *ITGB3*, encoding β3 integrin, is another HoxA10 target gene. In the current studies, we found activation of *ITGB3* transcription in Mll-Ell^+^ myeloid progenitor cells via HoxA9 and HoxA10. Increased expression of αvβ3 integrin increased Syk-activation; contributing to cytokine hypersensitivity. However, inhibiting Fgf-R partly reversed αvβ3 activity in Mll-Ell^+^ progenitor cells by decreasing *ITGB3* promoter activity in a βcatenin- and Cdx4-dependent manner. Inhibitors of Fgf-R or Syk impaired proliferation of CD34^+^ bone marrow cells from AML subjects with increased Hox-expression; with a greater combined effect. These studies identified a rational therapeutic approach to this AML subtype.

## INTRODUCTION

Hox proteins are highly conserved homeodomain (HD) transcription factors [1]. During embryogenesis, these proteins are expressed spatially with Hox1-4 expressed in head structures and Hox7-11 in abdominal/pelvic organs. During definitive hematopoiesis, Hox expression is temporal with Hox1-4 expressed in hematopoietic stem cells (HSC) and Hox7-11 in committed progenitors [2]. Consistent with this, *HOXA9*^−/−^ or *HOXA10*^−/−^ mice have aberrant development of genito-urinary structures and limited fertility [3, 4]. Although steady state hematopoiesis is relatively normal in these animals, *HOXA9*^−/−^ mice fail to develop granulocytosis in response to administration of exogenous G-CSF and *HOXA10*^−/−^ mice are unable to terminate emergency (stress) granulopoiesis [5, 6]. In the converse experiment, overexpression of HoxA9 or HoxA10 in murine bone marrow expands the committed myeloid progenitor population *in vitro* or *in vivo* [7–11]. Mice transplanted with HoxA10 overexpressing bone marrow develop granulocytosis that evolves to acute myeloid leukemia (AML) over time [11, 12]. Similar results are observed in mice transplanted with bone marrow co-overexpressing HoxA9 plus Meis1; a frequent Hox partner [13].

An adverse prognosis subset of human AML with increased expression of HD proteins, including HoxB4, A7-11, Cdx2, Cdx4 and Meis1, was previously defined. This includes leukemias with translocations or partial duplication of the *MLL1* gene (11q23 leukemia), *MYST3-CREBBP* translocation, or an adverse prognosis subset with normal cytogenetics [14–18]. Expression of various leukemia related *MLL1*-fusion proteins in murine bone marrow alters HD transcription factor expression, expands the myeloid progenitor population, and leads to myeloproliferation with progression to AML over time [19]. Additional studies identified recruitment of super-enhancer complexes to *HOX* genes by *MLL1*-fusion proteins as a mechanism for aberrant Hox expression in 11q23-AML [20, 21].

These studies imply HoxA9 and HoxA10 regulate proliferation and survival of bone marrow progenitor cells, but also influence progression of myelopoiesis. HoxA9 and HoxA10 have conserved DNA-binding HDs, but otherwise diverge. Consistent with this, we found a number of common target genes for the two Hox proteins, but differential regulation of some such genes. For example, HoxA9 and HoxA10 activate genes encoding Fibroblast growth factor 2 (Fgf2) and Transforming growth factor B2 (Tgfβ2) [22, 23]. However, genes encoding the phagocyte effectors gp91^*phox*^ and p67^*phox*^ are repressed by HoxA10 in progenitors, but activated by HoxA9 during myelopoiesis [24–26]. Conversely, HoxA9 represses, but HoxA10 activates, the Cdx4 and Triad1 genes [6, 27]. Cdx4 activates promoters of the *HOXA9* and *HOXA10* genes, creating a feedback mechanism among the three HD proteins [28–30].

The gene encoding β3 integrin (*ITGB3*) was identified as a HoxA10 target gene in endometrial cells [31]. In myeloid progenitor cells and differentiating phagocytes, we found HoxA10 activated *ITGB3* transcription and increased expression of αvβ3 integrin; enhancing β3 integrin dependent adhesion and Syk-activation in HoxA10 overexpressing cells [32]. In hematopoietic progenitor cells, αvβ3 integrin interacts with vitronectin (VN) in bone marrow niche and activates proliferative signals via Syk, Vav1, Rac1 and Pak1 [33, 34]. In mature phagocytes, αvβ3 mediates braking during vascular rolling via Syk, Vav1, Rho1 and Fak1 [35]. In the current studies, we hypothesize a mechanistic link between Hox dysregulation, αvβ3 integrin expression and activation of proliferative pathways in cells expressing *MLL1*-fusion proteins. Syk inhibitors are already in clinical trials and this connection would identify a subset of AML subjects for targeted testing of such agents.

We previously found that autocrine production of Fgf2 by Mll-Ell-expressing murine bone marrow progenitors activates Fgf-R signaling, thereby increasing cell proliferation and survival. Activation of Akt by Fgf2/Fgf-R results in Gsk3β-inactivation and stabilization of βcatenin protein. Increased βcatenin activity in Mll-Ell^+^ cells activates transcription of βcatenin target genes, including *CDX4* and *HOXA10*, in an Fgf2-dependent manner [22, 29, 30, 35]. Therefore, Mll-Ell increases expression of HoxA9 and HoxA10 directly, by interaction with their promoters, and indirectly via Fgf2, βcatenin and Cdx4. Non-canonical enhancement of αvβ3 signaling by Fgf-receptors was described in endothelial cells, but the for this was not defined [36]. In the current studies, we found that activation of Fgf-R enhanced αvβ3 integrin-induced Syk-activation in Mll-Ell^+^ bone marrow progenitor cells, and identified enhanced expression of β3 integrin as a mechanism for this cross regulation.

## RESULTS

### Mll-Ell increased ITGB3 transcription and β3 integrin mRNA expression via HoxA9 and HoxA10

**Figure 1:**
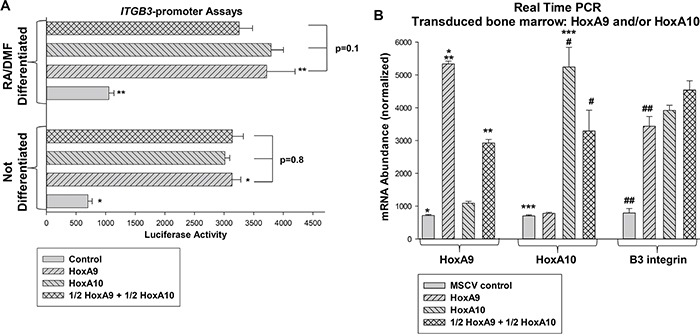
HoxA9 and HoxA10 cooperated to activate the *ITGB3* promoter and increased expression of β3 integrin **A.** HoxA9 and HoxA10 activated the same *ITGB3* cis element. U937 cells were co-transfected with a minimal promoter/reporter construct with or without three copies of the HoxA10-binding *ITGB3* cis element and vectors to overexpress HoxA9, HoxA10, HoxA9 + HoxA10 (total Hox vector constant) or empty vector. Reporter gene expression was determined with or without granulocyte differentiation with RA/DMF. Statistically significant differences (p<0.001, n=6) indicated by * or **. **B.** Overexpression of HoxA9 and HoxA10 increased expression of β3 integrin mRNA in myeloid progenitor cells. Murine bone marrow myeloid progenitor cells were transduced with a retroviral vector to express HoxA9, HoxA10, HoxA9 + HoxA10 (total Hox vector constant) or empty vector. Lin^−^CD34^+^ cells were analyzed by real time PCR as indicated. Statistically significant differences in mRNA expression (p<0.01, n=6) indicated by *, **, ***, #, or ##.

HoxA10 binds to and activates a cis element in the *ITGB3* promoter [31, 32]. Studies of other target genes suggested HoxA9 may regulate the same cis element, but might activate or repress. To investigate this, we co-transfected U937 myeloid cells with an artificial promoter/reporter construct containing three copies of the *ITGB3* cis element (or minimal promoter/reporter control vector) and vectors to overexpress HoxA9, HoxA10, HoxA9 + HoxA10 (maintaining a constant amount of Hox vector), or empty vector. We found equivalent activation of the *ITGB3* cis element by either Hox protein in U937 cells, with or without granulocyte differentiation (by retinoic acid/dimethyl formamide; RA/DMF) (Figure 1A). Activities of HoxA9 and HoxA10 were additive for promoter activation (Figure 1A).

To determine effects of HoxA9 on endogenous β3 integrin message, we performed studies with transduced murine bone marrow cells. For these experiments, bone marrow mononuclear cells were transduced with retroviral vectors to express HoxA9, HoxA10, HoxA9 + HoxA10, or empty MSCV control vector. As in the studies above, the total amount of Hox vector was constant. Cells were cultured in GM-CSF, IL3 and Scf followed by separation of Lin^−^CD34^+^ cells (referred to as myeloid progenitor conditions in these studies) with or without *ex vivo* differentiation with G-CSF [6, 11, 22, 23]. B3 integrin mRNA was quantified by real time PCR. Overexpressing these Hox proteins significantly increased β3 integrin mRNA (p<0.001, n=3) in an additive manner (Figure 1B).

**Figure 2:**
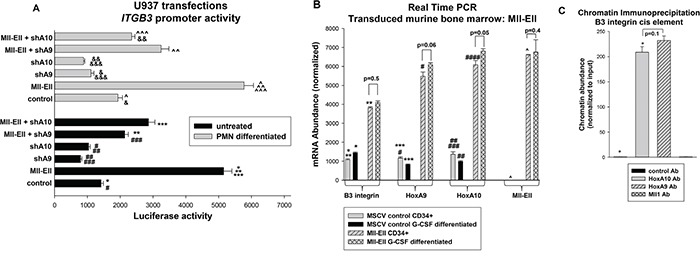
Mll-Ell increased *ITGB3* promoter activity and β3 integrin expression in a HoxA9/HoxA10-dependent manner **A.** Knockdown of HoxA9 or HoxA10 impaired *ITGB3* cis element activation by Mll-Ell. Cells were co-transfected with a minimal promoter/reporter construct with three copies of the *ITGB3* cis element (or control vector), vectors to express Mll-Ell (or control), and shRNAs specific to HoxA9 or HoxA10 (or scrambled controls). Reporter gene expression was determined with or without granulocyte differentiation with RA/DMF. Statistically significant differences (p<0.001, n=6) indicated by *, **, ***, #, ##, ###, &, &&, &&&, ^, ^^, or ^^^. **B.** Expression of Mll-Ell increased expression of β3 integrin in myeloid progenitor cells. Murine bone marrow myeloid progenitor cells were transduced with a retroviral vector to express Mll-Ell or empty control vector. Lin^−^CD34^+^ cells were analyzed by real time PCR as indicated. Statistically significant differences in mRNA expression (p<0.01, n=6) indicated by *, **, ***, #, ##, ###, or ^. **C.** HoxA9 and HoxA10 bound to the *ITGB3* promoter, but Mll-Ell did not. U937 chromatin was co-immuno-precipitated with antibodies to HoxA9, HoxA10, Mll1 or irrelevant control antibody and amplified with primers flanking the *ITGB3* cis element. Statistically significant difference (p<0.0001, n=3) indicated by *.

We next investigated the impact of the Mll-Ell on *ITGB3* transcription in U937 transfection experiments, similar to those above. We found that co-transfection with an Mll-Ell expression vector increased activity of the *ITGB3* cis element in comparison to control, with or without granulocyte differentiation (p<0.001, n=6) (Figure 2A). To determine if this was dependent on HoxA9 or HoxA10, we co-transfected cells with the *ITGB3* cis element reporter and vectors to express Mll-Ell plus shRNAs specific to HoxA9 or HoxA10 (or scrambled shRNA control). We found either specific shRNA significantly decreased the effect of Mll-Ell on the *ITGB3* cis element (~50% reduction; p<0.001, n=6) (Figure 1B). In undifferentiated transfectants, the effect of HoxA9-shRNA was slightly greater than HoxA10-shRNA and vice versa for differentiated transfectants.

To investigate the impact of Mll-Ell on endogenous β3 integrin mRNA, we transduced murine bone marrow cells with an Mll-Ell retroviral expression vector or empty control vector. We found significantly increased abundance of β3 integrin mRNA in Mll-Ell expressing cells, with or without G-CSF-differentiation (p<0.001, n=3) (Figure 2B). Expression of HoxA9 and HoxA10 was increased in these cells, relative to control, as anticipated (p<0.001, n=3).

We also investigated the *ITGB3* cis element for interaction with HoxA9 or Mll-Ell by chromatin immuno-precipitation. Co-precipitating chromatin was amplified by quantitative real time PCR. HoxA10 antibody or an irrelevant antibody were positive and negative controls, respectively. We found specific interaction of both HoxA9 and HoxA10 with the cis element, but not Mll-Ell (Figure 2C). In additional control studies, none of these antibodies co-precipitated intron 1 of this gene (not shown).

### Mll-Ell increased activation of β3 integrin and Syk in primary murine bone marrow cells

**Figure 3:**
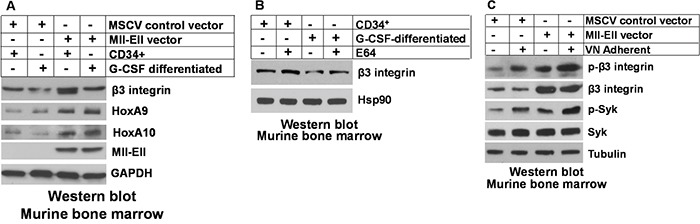
Mll-Ell increased β3 integrin protein in murine myeloid progenitors and differentiating granulocytes **A.** Mll-Ell increased expression of β3 integrin protein in myeloid progenitor cells, but had less effect on granulocytes. Murine bone marrow myeloid progenitor cells were transduced with a retroviral vector to express Mll-Ell or empty control vector. Western blots of Lin^−^CD34^+^ cell lysates were serially probed +/− G-CSF-differentiation as indicated. **B.** Treatment with a lysosomal inhibitor increased β3 integrin protein in differentiating granulocytes. Cells were analyzed after treatment with a lysosomal stabilizing agent (E64) versus control. Western blots were serially probed as indicated. **C.** Adhesion of Mll-Ell-expressing myeloid progenitor cells to vitronectin increased activation of β3 integrin and Syk. Cells were assayed +/− adherence to vitronectin coated plates. Lysates were analyzed by serially probed Western blots as indicated.

We next explored the impact of Mll-Ell on β3 integrin protein expression and activation of downstream pathways in the transduced murine bone marrow cells, described above. We found increased abundance of β3 integrin protein in Mll-Ell-expressing myeloid progenitor cells relative to control (Figure 3A). In contrast to our findings for mRNA expression, G-CSF-differentiation resulted in a relative decrease in abundance of β3 integrin protein in both Mll-Ell^+^ and control cells (Figure 3A). This discrepancy suggested differentiation might de-stabilize β3 integrin protein. In control experiments, Mll-Ell-expression increased HoxA9 and HoxA10 protein (Figure 3A), with or without differentiation, as expected.

In myeloid progenitors and granulocytes, β3 integrin exists as an αvβ3 dimer. Metabolism of this dimer is regulated in part by ubiquitination of αv followed by αvβ3 internalization and degradation in the lysosome [41]. Ubiquitination and lysosomal degradation of β3 has also been described [42]. We investigated the possibility that G-CSF-differentiation enhanced lysosomal degradation of β3 integrin. For these studies, cells were treated with a lysosomal stabilizing agent (E64 versus control) and lysates analyzed for β3 integrin by Western blot. We found treatment with E64 reversed the effect of G-CSF of β3 integrin protein abundance (Figure 3B; note equivalence of first and fourth lanes).

We previously determined that HoxA10 overexpression enhanced VN-induced activation (phosphorylation) of β3 integrin and Syk kinase (a downstream intermediate) versus control cells [32]. In the current study, we investigated VN-induced activation of β3 integrin and Syk in Mll-Ell^+^ cells. We found relatively more activated β3 integrin and Syk in VN adherent Mll-Ell^+^ murine myeloid progenitor cells versus non-adherent cells, and in Mll-Ell^+^ versus control cells (Figure 3C).

### Inhibition of Fgf-R in Mll-Ell^+^ progenitor cells decreased β3 integrin expression and αvβ3 signaling

**Figure 4:**
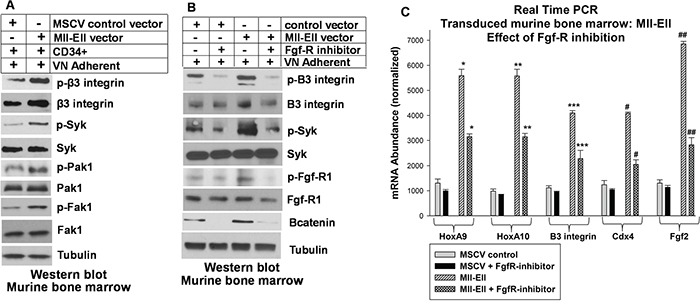
Inhibiting the Fgf-receptor in Mll-Ell-transduced myeloid progenitor cells decreased β3 integrin expression and β3 integrin/Syk-activation by vitronectin Murine bone marrow myeloid progenitor cells were transduced with a vector to express Mll-Ell or control vector. Lin^−^CD34^+^ cells were analyzed. **A.** Mll-Ell-expression increased activation of β3 integrin, Syk, Fak1 and Pak1 in vitronectin adherent cells. Cells were analyzed by serially probing Western blots for total and phospho (activated) proteins. **B.** Fgf-R inhibition decreased activation of β3 integrin and Syk in vitronectin adherent cells, and decreased β3 integrin protein in Mll-Ell^+^ cells. Some cells were treated with an Fgf-R inhibitor (PD173074) and lysates were analyzed by Western blots serially probed for phospho/total proteins. Decreased phospho-Fgf-R and total βcatenin were positive controls for Fgf-R inhibition. **C.** Fgf-R inhibitor treatment decreased mRNA for Cdx4 (βcatenin-target-gene), HoxA9 and HoxA10 (Cdx4-target-genes), β3 integrin and Fgf2 (HoxA9/HoxA10-target-genes). Cells were analyzed for mRNA by real time PCR. Statistically significant differences (p<0.001, n=6) indicated by *, **, ***, #, or ##.

Since both increased β3 integrin expression and autocrine production of Fgf2 are present in Mll-Ell^+^ bone marrow progenitor cells, we investigated functional interaction between these receptors. First, we investigated the impact of Mll-Ell on signaling pathways downstream from VN-induced αvβ3/Syk-activation. For these studies, murine bone marrow cells were transduced with retroviral vector to express Mll-Ell or control vector and VN-adherent myeloid progenitors were assayed by Western blot. Activation of Syk by αvβ3 integrin results in activation of Vav1 and subsequently Rac1/Pak1 or Rho1/Fak1. We found enhanced VN-induced activation of these pathways in Mll-Ell^+^ versus control cells (Figure 4A).

We next investigated the impact of inhibiting Fgf-R on the β3 integrin signaling in Mll-Ell^+^ or control murine myeloid progenitor cells. For these studies, we treated VN adherent cells with an Fgf-R inhibitor (PD173074 versus control). We found Fgf-R inhibition decreased activation (phosphorylation) of β3 integrin and Syk in VN-adherent Mll-Ell^+^ cells and control cells (Figure 4B). We also noted that Fgf-R-inhibitor treatment decreased abundance of β3 integrin protein in Mll-Ell^+^ cells (Figure 4B). As expected, PD173074 decreased Fgf-R1-phosphorylation (activation) and βcatenin protein (Figure 4B).

These results suggested Fgf-R influenced activation of αvβ3 integrin in Mll-Ell^+^ myeloid cells by increasing β3 integrin expression. Consistent with this, we found that Fgf-R inhibition also decreased expression of β3 integrin mRNA in the transduced cells, with a greater effect in Mll-Ell^+^ cells versus control (~40% decrease versus ~10%) (Figure 4C). Bcatenin activates the *CDX4* and *HOXA10* promoters, and Cdx4 activates transcription of *HOXA9* and *HOX10* genes [28–30]. We hypothesized that Fgf-R inhibition destabilized βcatenin protein, especially in Mll-Ell^+^ cells with autocrine Fgf2 production. This would decrease expression of βcatenin target genes including *CDX4*, Cdx4 target genes including *HOXA9* and *HOXA10*, and HoxA9 and HoxA10 target genes including *ITGB3* and *FGF2*. Consistent with this, expression of mRNA representing these HD proteins, β3 integrin and Fgf2 was significantly decreased by Fgf-R inhibitor treatment of Mll-Ell^+^ myeloid progenitor cells (Figure 4C).

**Figure 5:**
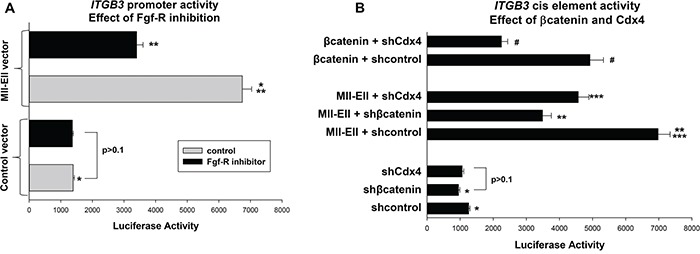
The Fgf-receptor influenced *ITGB3* promoter activity in a βcatenin- and Cdx4-dependent manner U937 cells were transfected with the *ITGB3* cis element construct (or control vector) and analyzed for reporter activity. **A.** Fgf-receptor-inhibition decreased *ITGB3* promoter activity in Mll-Ell-expressing cells. Cells were co-transfected with a vector to express Mll-Ell (or control vector) and reporter assays performed +/− PD173074. Statistically significant differences indicated by * or ** (p<0.0001, n=6). **B.** βcatenin increased *ITGB3* cis element activity in a Cdx4-dependent manner, and Mll-Ell increased cis element activity in a βcatenin- and Cdx4-dependent manner. Cells were co-transfected with vector to express βcatenin or Mll-Ell (or vector control) plus an shRNA specific to Cdx4 or βcatenin (or scrambled control). Statistically significant differences (p<0.001, n=6) indicated by *, **, ***, or #.

We explored this mechanism at the level of *ITGB3* promoter activity in U937 transfection studies. We first tested the effect of Fgf-R inhibition on the Hox-binding *ITGB3* cis element using the promoter/reporter construct described above. Cells were co-transfected with an Mll-Ell expression vector or control. We found that treatment with PD173074 significantly decreased *ITGB3*-cis element activity in Mll-Ell transfectants (p<0.001, n=3), but had little effect on the cis element in control transfectants (p=0.8, n=3) (Figure 5A). These results were concordant with effects on endogenous β3 integrin mRNA, above.

To investigate roles for βcatenin and Cdx4 in this process, we co-transfected U937 cells with the *ITGB3* cis element/reporter construct and various combinations of vectors to express Mll-Ell (or control vector) or knockdown βcatenin or Cdx4 (with specific shRNAs versus scrambled, control shRNAs). We found that knockdown of either βcatenin or Cdx4 significantly decreased *ITGB3* cis element activity in Mll-Ell expressing transfectants (p<0.0001, n=6) (Figure 5B). Knockdown of these proteins also decreased *ITGB3* cis element activity in control transfectants, but to a lesser extent (~10% versus ~40% for Mll-Ell^+^ cells). These results indicated activation of *ITGB3* transcription by Mll-Ell was influenced by βcatenin and Cdx4. To determine if the effect of βcatenin required Cdx4 (as per our hypothesis) we performed additional studies. U937 cells were co-transfected with the *ITGB3* cis element reporter construct, a vector to express βcatenin (or control vector), and a vector to express a Cdx4 specific shRNA (or scrambled control). We found that overexpression of βcatenin increased *ITGB3* promoter activity, but this effect was almost completely abolished by knockdown of Cdx4 (Figure 5B).

### Fgf-R-inhibition influenced adhesion and Syk-inhibition influenced cytokine hypersensitivity in Mll-Ell^+^ myeloid progenitor cells

**Figure 6:**
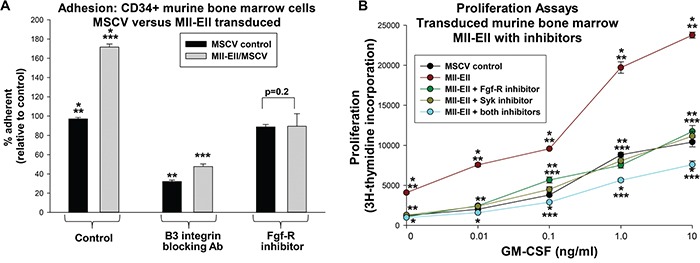
Mll-Ell increased adhesion and proliferation of murine myeloid progenitor cells Cells were transduced with a retroviral vector to express Mll-Ell or control vector. **A.** Mll-Ell increased β3 integrin-dependent adhesion of myeloid progenitor cells, but this was decreased by Fgf-R inhibition. Some cells were analyzed for % vitronectin adhesion after treatment with a β3 integrin blocking antibody or PD173074. Statistically significant differences (p<0.0001, n=6) indicated by *, ** or ***. **B.** Fgf-receptor inhibition cooperated with Syk inhibition to decrease cytokine hypersensitivity of Mll-Ell^+^ myeloid progenitor cells. Cells were stimulated with a dose titration of GM-CSF and proliferation assayed by ^3^H-thymidine incorporation. Cells treated with PD173074 (Fgf-R inhibitor), BAY-3606 (Syk inhibitor), both or neither were analyzed. Statistically significant differences (p<0.01, n=6) indicated by *, ** or *** for a given cytokine dose.

We performed adhesion assays to determine the functional consequences of decreased β3 integrin in Mll-Ell^+^ cells treated with Fgf-R inhibitor. We analyzed Mll-Ell or control vector transduced, Lin^−^CD34^+^ murine bone marrow myeloid progenitor cells for adhesion to VN. We found a significantly greater percent of Mll-Ell^+^ cells adherent to VN versus control cells (p<0.001, n=3) (Figure 6A). Treatment of Mll-Ell^+^ cells with Fgf-R inhibitor decreased the percent VN-adherent cells so that was not different than control (p=0.2, n=3) (Figure 6A). This was consistent with a decrease in β3 integrin protein in Fgf-R inhibitor treated, Mll-Ell^+^ cells. Dependence of adhesion on β3 integrin was demonstrated by pre-incubation with a β3 blocking antibody (p<0.001, n=3 with versus without antibody).

We also investigated the impact of inhibiting Syk, Fgf-R, or both on proliferation of Mll-Ell-transduced cells. In myeloid progenitor cells, Mll-Ell confers an increased proliferative response to cytokines, including GM-CSF (i.e. cytokine hypersensitivity) [35]. We assayed Mll-Ell^+^ or control vector transduced murine myeloid progenitor cells for ^3^H thymidine incorporation. We found treatment of Mll-Ell^+^ cells with Syk inhibitor (BAY-3606) significantly decreased proliferation at all GM-CSF doses (p<0.01, n=3) (Figure 6B). Fgf-R inhibitor (PD173074) also decreased cytokine-induced proliferation in these cells, and the effect of the two together was significantly greater than either alone (p<0.01, n=3) (Figure 6B). Proliferation of control vector transduced cells was not influenced by inhibitor treatment (p=0.2, n=3) (not shown).

### Expression of HoxA9 and HoxA10 in human CD34^+^ AML cells correlated with sensitivity to inhibition of Fgf-R or Syk

Based on the studies above, we investigated the impact of inhibiting Fgf-R or Syk-kinase activity on proliferation of human AML cells. For these studies, we compared CD34^+^ bone marrow cells from AML subjects to CD34^+^ bone marrow cells from control individuals. Since increased Hox expression was previously identified in clinical situations in addition to 11q23-AML, we first analyzed cells for expression of HoxA9 and HoxA10 mRNA [14–18].

**Figure 7:**
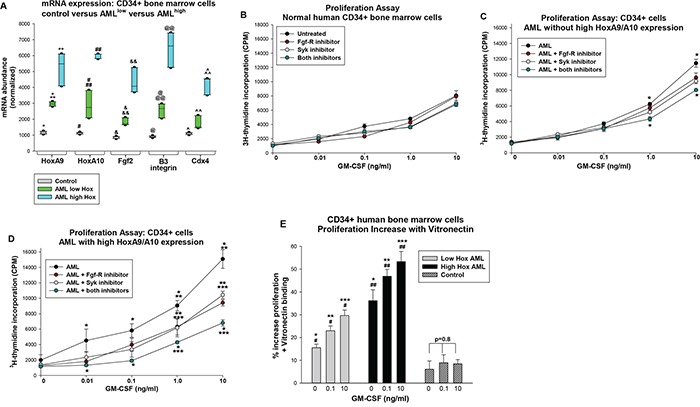
Expression of HoxA9 and HoxA10 in human AML cells correlated with sensitivity to inhibition of Fgf-receptor and/or Syk kinase Lin^−^CD34^+^ bone marrow cells from human AML subjects or normal donors were compared. **A.** AML samples were grouped according to HoxA9 and HoxA10 expression. Gene expression was determined by real time PCR. Statistically significant differences (p<0.01, n=3 for control and n=6 for AML samples) indicated by *, **, #, ##, &, &&, @, @@, ^, or ^^. **B.** GM-CSF induced proliferation of control CD34^+^ cells was not influenced by inhibition of Fgf-receptor or Syk kinase. Control cells were analyzed for proliferation in response to a dose titration of GM-CSF in the presence of Fgf-receptor inhibitor, Syk inhibitor, both or neither. 3H-thymidine incorporation was not significantly different between the four groups at any cytokine dose (p>0.05, n=3) **C.** Combined treatment with Syk plus Fgf-receptor inhibitors decreased proliferation of low-Hox expressing AML at high GM-CSF doses. Cells from the low-Hox expressing AML group were analyzed as above. Statistically significant differences between untreated and Fgf-R-inhibitor + Syk-inhibitor treated cells (p<0.001, n=6) indicated by * for a given cytokine dose. **D.** GM-CSF induced proliferation of cells from the high-Hox expressing AML group was decreased by treatment with Fgf-receptor inhibitor or Syk inhibitor and further decreased with the combination. Cells from high-Hox expressing AML were analyzed as above. Statistically significant differences (p<0.001, n=6) between untreated versus Fgf-R-inhibitor + Syk-inhibitor treated indicated by *, untreated versus Fgf-R-inhibitor or Syk-inhibitor treated by **, and Fgf-R-inhibitor or Syk-inhibitor treated versus Fgf-R-inhibitor + Syk-inhibitor treated by *** for a given cytokine dose. **E.** GM-CSF induced proliferation of cells from the high-Hox expressing AML group was increased by vitronectin binding. Control, low-Hox and high-Hox expressing CD34+ cells were analyzed for proliferation as above with versus without vitronectin binding. Results were normalized for cell number. Statistically significant differences (p<0.02) between the groups is indicated by *, **, or ***and for different GM-CSF doses within a group by # or ##.

AML samples fell into two groups; in the first group expression of HoxA9 averaged 2.5 standard deviations and HoxA10 5.7 standard deviations above control cells (n=6 AML samples and n=3 normal CD34^+^ samples), and in the second group expression averaged 6.5 and 13.5 standard deviations, respectively, above control (n=6 AML samples) (Figure 7A). We found greater expression of Fgf2, β3 integrin and Cdx4 mRNA in the low-Hox group versus control, but expression in the high-Hox group was significantly greater than control or low-Hox groups (p<0.0001 for all comparisons, n=3 control samples and n=6 AML samples) (Figure 7A). The three groups passed an equal variance of means test for expression of the messages (p>0.1).

We performed proliferation assays to determine if this grouping predicted response to inhibition of Fgf-R, Syk or both. Studies of control human CD34^+^ bone marrow cells indicated a dose dependent response to GM-CSF that was not significantly altered by PD173074, BAY-3603 or both for any cytokine dose (p>0.1, n=3) (Figure 7B). In the low-Hox AML group, the proliferation curve was shifted up relative to control cells and this was most pronounced at highest GM-CSF doses (p<0.001, n=6) (Figure 7C). Proliferation in the low-Hox group was not significantly altered by inhibition of Fgf-R or Syk alone, but was decreased by the combination at the two highest cytokine doses (p<0.001, n=6) (Figure 7C). Proliferation of the high-Hox expressing group was significantly greater at most doses of GM-CSF compared to control CD34^+^ cells or the low-Hox AML group (p<0.01, n=6) (Figure 7D). For the high-Hox group, proliferation was significantly decreased by inhibition of either Fgf-R or Syk at the highest GM-CSF doses (p<0.001, n=6) (Figure 7D). However, the combined effect of the two inhibitors significantly decreased proliferation of high-Hox expressing CD34^+^ cells at all tested cytokine doses (p<0.001, n=6 for comparison to no treatment) (Figure 7D).

If the increase in αvβ3 integrin expression on high-Hox expressing cells is functionally significant, we would anticipate augmented proliferation of these cells upon VN-binding in comparison to low-Hox expressing or control CD34^+^ cells. To test this, CD34^+^ cells were analyzed in proliferation assays with versus without VN binding. Results were normalized for cell numbers. In control cells, we found that VN binding increased proliferation by less than 10% and this was not GM-CSF-dose dependent (p=0.78, n=4). We found that VN binding increased proliferation of AML samples significantly more than control, and the effect was significantly greater in the high-Hox group (p<0.02 for low versus high-Hox expression). For both AML groups, the effect of VN on proliferation increased with GM-CSF dose (p<0.001, n=3 for low-Hox AML; p<0.01, n=5 for high-Hox AML). Therefore, VN-binding had a greater effect on cytokine hypersensitivity in the high-Hox group.

## DISCUSSION

*ITGB3* was initially identified as a target gene for HoxA10 in studies with endometrial cells [31]. We subsequently found that HoxA10 activated the *ITGB3* promoter in myeloid progenitors and differentiating phagocytes [32]. Ligand binding to αvβ3 integrin transduces signals from Syk to Vav1. In HSC and myeloid progenitors, this results in activation of Rac1/Pak1 and transduction of proliferative signals [33, 34]. In mature phagocytes, Rho/Fak1 are activated and αvβ3 participates in phagocyte rolling along the vascular endothelium [35]. Recent siRNA screening studies determined that β3 integrin-knockdown impaired leukemic transformation by Mll-Af9 fusion proteins in a murine model [43]. These studies did not connect the observations to induction of Hox-expression by *MLL1*-fusion proteins nor the established role of HoxA10 in *ITGB3* transcription. Since no mechanism was identified, it was unknown if Mll-Af9 influenced β3 integrin expression, or if β3 integrin was required for cooperation with another Mll-Af9 regulated event. Alternatively, there might be a general requirement for αvβ3 for interaction of leukemia stem cells (LSCs) with the bone marrow niche. In the current work, we found *ITGB3* was activated by Mll-Ell in a HoxA9 and HoxA10-dependent manner. This was similar to regulation of *FGF2* and *TGFB2*; genes that promote progenitor expansion, but also enhance phagocyte effector functions [22, 23]. Most importantly, our studies demonstrate that increased Hox-expression in human CD34^+^ AML cells correlated with a response to inhibition of Fgf-R or Syk in proliferation assays.

**Figure 8:**
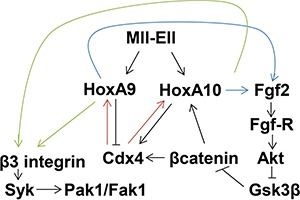
Schematic representation of cross regulation between β3 integrin and Fgf2 in Mll-Ell-expressing myeloid progenitor cells Arrows indicate increased expression/activity and ⊣ represents inhibition of expression/activity.

Activation of *ITGB3* transcription by HoxA9/HoxA10 was not specific to differentiation stage, suggesting that cytokine-induced tyrosine phosphorylation of HoxA9 or HoxA10 was not required. Consistent with this, we found no difference in *ITGB3* cis element activation with tyrosine mutant forms of HoxA9 or HoxA10 (not shown). This is similar to co-activation of *FGF2* and *TGFB2* promoters by these Hox proteins. In contrast, target genes activated by HoxA9 but repressed by HoxA10 (or vice versa) are regulated in a differentiation stage specific manner by the relative affinities of phosphorylated HoxA9 versus HoxA10 for cis element-binding. Examples include the phagocyte effector genes *NCF2* and *CYBB*, the E3 ubiquitin ligase gene *ARIH2*, and *CDX4* [24–28]. In the latter case, HoxA10 activates *CDX4* transcription in HSC and myeloid progenitor cells, but HoxA9 represses *CDX4* during myelopoiesis (Figure 8). These three HD proteins cross regulate, since Cdx4 activates specific cis elements in the *HOXA9* and *HOXA10* genes, preferentially in bone marrow progenitor cells.

Our current studies identified an additional set of cross regulatory interactions for HoxA9, HoxA10 and the target genes *FGF2* and *ITGB3* (Figure 8). We found that Fgf-R-inhibition decreased αvβ3-dependent VN-adhesion and Syk activation. Further investigation determined that Fgf2/Fgf-R-signaling increased β3 integrin expression in Mll-Ell^+^ cells. Fgf2/Fgf-R-activation stabilizes βcatenin, increasing expression of βcatenin target genes, including *CDX4* and *HOXA10*. The *HOXA9* and *HOXA10* promoters are activated by Cdx4, and we found Fgf-R inhibition was also associated with decreased HoxA9 and HoxA10 expression in Mll-Ell^+^ bone marrow progenitor cells. We additionally found βcatenin/Cdx4-dependent impairment of *ITGB3* promoter activation in Fgf-R inhibitor treated, Mll-Ell^+^ cells.

The cross regulation between HD proteins, growth factors and integrins we identify here suggests it should be possible to identify a subset of AML subjects who would respond to inhibition of Fgf-R, Syk or both. We tested the possibility that expression levels of HoxA9, HoxA10 and Cdx4 predicted this response. We found that the subset of CD34^+^ cells from AML subjects with highest expression of these HD proteins expressed the most Fgf2 and β3 integrin, and responded to treatment with Fgf-R or Syk inhibitors with decreased proliferation. The effect of these two inhibitors were at least additive in these cells. These results suggest successful targeting with these agents might be predicted based on these molecular markers.

## MATERIALS AND METHODS

### Ethics statement

Investigation has been conducted in accordance with the ethical standards and according to the Declaration of Helsinki and according to national and international guidelines and has been approved by the authors' institutional review board.

### Plasmid vectors

The cDNA for human HoxA10 was obtained from C. Largman (University of California, San Francisco) [37]. The HoxA9 cDNA was generated by reverse transcription and PCR from U937 cells, as described [26]. These cDNAs were subcloned into the mammalian expression vector pcDNAamp (Invitrogen, Carlsbad, CA) and the murine retroviral vector pMSCVpuro (Clontech, Mountain View, CA), as described [32, 38]. A vector with the Mll-Ell fusion protein was obtained from D.E. Zhang (University of California, San Diego). HoxA10, HoxA9 and Cdx4 specific shRNAs and scrambled control sequences were designed using the Promega website (Promega, Madison, WI) and subcloned into the pLKO.1puro vector (from Dr. Kathy Rundell, Northwestern University, Chicago). Several sequences were tested and the most efficient combined. The βcatenin cDNA was obtained from Addgene (Cambridge MA) and subcloned into the pcDNA3 vector.

A synthetic oligonucleotide with the Hox-binding region of the human *ITGB3* promoter was multimerized, as previously described, and subcloned into the pGL3-promoter reporter vector (Promega, Madison, WI) [32].

### Oligonucleotides

Oligonucleotides were custom synthesized by MWG Biotech (Piedmont, NC). These oligonucleotides represent Hox-binding sequence from the *ITGB3* promoter; −1973 to −1933 bp sequence (5-GGGGGGCTTATAATGTTATTTTTAGTTTACAGGTTCTTAC-3) [32]. Primers for ChIP amplification of the *ITGB3* promoter were: 5′-TAACTTTGGAATGCCCTTGG-3′ and 5′-CTACACCTCGTTTGCGTGTG-3′. Control sequences for these studies were from *ITGB3* intron 1: 5′-GCTGTAGCTTCCTGGGTGAG-3′ and 5′-CATCCTGCTCCAAAACAACC-3′.

### Myeloid cell line culture

The human myelomonocytic leukemia cell line U937 [39] was obtained from A. Kraft (University of Arizona Comprehensive Cancer Center, Phoenix, AZ). Cells were maintained and differentiated (with retinoic acid (RA) and dimethylformamide (DMF)) as described [39].

### Primary murine bone marrow studies

Animal studies were performed according to protocols approved by the Animal Care and Use Committees of Northwestern University and Jesse Brown VA.

Mice were euthanized according to institutional guidelines. Bone marrow mononuclear cells were obtained from the femurs of C57/BL6 mice. Bi-potential granulocyte/monocyte progenitor cells were cultured (2 × 10^5^ cells/ml) for 48 hrs in DME media supplemented with 10% fetal calf serum, 1% pen-strep, 10 ng/ml murine GM-CSF (R and Diagnostics Systems Inc., Minneapolis, MN), 10 ng/ml murine recombinant IL-3 (R & D Systems Inc.) and 100 ng/ml SCF (R & D Systems Inc.) followed by separation of Lin^−^CD34^+^ cells using the Miltenyi magnetic bead system (Miltenyi Biotechnology, Auburn, CA) (referred to as myeloid progenitor cells) [11, 23, 26]. Some cells were differentiated in DME supplemented with 10% fetal calf serum, 1% pen-strep, 20 ng/ml G-CSF (R & D Systems Inc.) and 10 ng/ml IL3.

Retrovirus was generated with Mll-Ell/MSCV, HoxA9/MSCV, HoxA10/MSCV or control MSCV plasmid using the Phoenix cell packaging line according to manufacturer's instructions (Stratagene, La Jolla, CA). The average viral concentration was 10^7^ pfu/ml. Bone marrow mononuclear cells were cultured for 24 hrs in 10 ng/ml GM-CSF, 10 ng/ml IL3 and 100 ng/ml SCF. Cells were transduced by incubation with retroviral supernatant supplemented with polybrene (6 μg/ml) as described [11, 22, 25]. Transduced cells were selected for 48 hrs in puromycin, followed by culture lineage depletion and CD34 selection (as above). Transgene expression was confirmed by real time PCR and Western blot.

### Human leukemia cells

Human studies were performed with the approval of the Northwestern University IRB. Bone marrow was obtained from leukemia subjects at the time of diagnostic evaluation. Control CD34^+^ bone marrow cells were purchased from Stem Cell Technologies (Vancouver, Canada). Control and leukemia bone marrow samples were processed in the same manner prior to analysis. Lin^−^CD34^+^ cells were isolated (using the Miltenyi magnetic bead system) and cultured for 24 hrs in human GM-CSF, IL3 and Scf (at concentrations indicated above). For gene expression and proliferation studies, all experiments were performed in triplicate for each independent sample, the replicates for each sample were averaged, and that average was used in analysis the various groups.

### Cell adhesion assays

Dishes were coated overnight at 4°C with 20 μg/ml vitronectin. Transduced murine bone marrow cells were counted and transferred to vitronectin coated dishes. Cells were incubated for 16 h at 37°C, 5% CO2. In some experiments, cells were pre-incubated with 20 μg/ml of antibody to β3 integrin or irrelevant control antibody (Chemicon, Temecula, CA), or 100 nM of PD173074 (R & D Systems. Inc.). The dishes were washed with phosphate-buffered saline, and adherent cells were fixed in 3.7% formaldehyde, stained with 0.1% crystal violet, solubilized in 10% acetic acid and absorbance at 540 nm was determined [32].

### Quantitative real time PCR

RNA was isolated using Triazol reagent (Gibco-BRL, Gaithersburg MD). Primers were designed with Applied Biosystems software. Real time PCR was performed with SYBR green according to the “standard curve” method. Result were normalized to 18S and actin (for mRNA determination) or total input chromatin (for chromatin immuno-precipitation studies).

### Chromatin immuno-precipitation

Cells were incubated briefly in media supplemented with formaldehyde to generate DNA-protein cross links. Lysates were sonicated to generate chromatin fragments with an average size of 200 bp and underwent one round of immuno-precipitation with antibodies to HoxA9, HoxA10, Mll1 or irrelevant antibody [22, 27, 40]. HoxA9 and HoxA10 antibodies are not cross reactive and do not prevent DNA-binding (N-20 and A-20 from Santa Cruz Biotechnology, Santa Cruz CA). The Mll1 antibody also does not prevent DNA binding (Abcam Technologies, Cambridge MA). Irrelevant antibody was to glutathione-s-transferase (anti-GST antibody, Santa Cruz Biotechnology). Chromatin was amplified by real time PCR with sets of primers flanking the previously identified Hox-binding cis elements in the *ITGB3* promoter, or with primers representing irrelevant regions in exon 1, as previously described [32].

### Myeloid cell line transfections and assays

U937 cells were co-transfected with a construct with three copies of the HoxA10-binding *ITGB3* promoter cis element linked to a minimal promoter and Luciferase reporter (or minimal promoter/reporter control) and vectors to express various combinations of Mll-Ell, HoxA9, HoxA10, HoxA9 specific shRNAs, HoxA10 specific shRNAs or appropriate control vectors. Cells were also transfected with a β-galactosidase reporter vector to control for transfection efficiency (CMVβ-gal). Transfectants were analyzed with or without granulocyte differentiation with RA + DMF.

### Western blots

Cells were lysed by boiling in 2X SDS sample buffer. Lysate proteins were separated by SDS-PAGE and transferred to nitrocellulose. Western blots were serially probed with antibodies to various proteins, including a loading control. Each experiment was repeated at least three times with different batches of lysate proteins. Representative blots are shown.

### Cell proliferation assays

Bone marrow cells were cultured in 10 ng/ml GM-CSF, 10 ng/ml IL3, 100 ng/ml SCF, deprived of cytokines for 24 hrs (in DME with 10% FCS), and stimulated for 24 hrs with a dose titration of GM-CSF (0.01 to 10 ng/ml). Some cells were incubated with Fgf-R1 inhibitor (PD173074; 100 nM), Syk inhibitor (BAY-3606; 50 nM) (Apex Biotechnology, Houston TX), both or buffer control. Cell proliferation was determined by uptake of ^3^H-thymidine [22, 35]. Some samples were assayed after binding to vitronectin. For these studies, proliferation of adherent versus non-adherent samples were normalized for cell number and percent increase in proliferation with VN-adhesion was calculated.

### Statistical analysis

Statistical significance was determined by Student's t-test and ANOVA using SigmaPlot software. Graphs are presented with error bars representing standard error calculations. P values of <0.02 were considered statistically significant.
